# Amplification of *cox2* (∼620 bp) from 2 mg of Up to 129 Years Old Herbarium Specimens, Comparing 19 Extraction Methods and 15 Polymerases

**DOI:** 10.1371/journal.pone.0003584

**Published:** 2008-10-31

**Authors:** Sabine Telle, Marco Thines

**Affiliations:** University of Hohenheim, Institute of Botany 210, Stuttgart, Germany; American Museum of Natural History, United States of America

## Abstract

During the past years an increasing number of studies have focussed on the use of herbarium specimens for molecular phylogenetic investigations and several comparative studies have been published. However, in the studies reported so far usually rather large amounts of material (typically around 100 mg) were sampled for DNA extraction. This equals an amount roughly equivalent to 8 cm^2^ of a medium thick leaf. For investigating the phylogeny of plant pathogens, such large amounts of tissue are usually not available or would irretrievably damage the specimens. Through systematic comparison of 19 DNA extraction protocols applied to only 2 mg of infected leaf tissue and testing 15 different DNA polymerases, we could successfully amplify a mitochondrial DNA region (*cox2*; ∼620 bp) from herbarium specimens well over a hundred years old. We conclude that DNA extraction and the choice of DNA polymerase are crucial factors for successful PCR amplification from small samples of historic herbarium specimens. Through a combination of suitable DNA extraction protocols and DNA polymerases, only a fraction of the preserved plant material commonly used is necessary for successful PCR amplification. This facilitates the potential use of a far larger number of preserved specimens for molecular phylogenetic investigation and provides access to a wealth of genetic information in preserved in specimens deposited in herbaria around the world without reducing their scientific or historical value.

## Introduction

Millions of herbarium specimens are deposited in herbaria around the world. The primary aim of these institutions is the permanent conservation of a diverse sample of plants, algae and fungi for documentation and comparative investigations, particularly of a taxonomic nature. With the advent of PCR [1] and cheap sequencing techniques [2], molecular phylogenetic investigations have been the most important source of phylogenetic data and an important touchstone for morphology based taxonomies. The wealth of herbarium specimens preserved in international herbaria has, however, scarcely been exploited in this context, although already Bruns et al. [3] addressed the topic of extracting DNA from fungal herbarium specimens. The main reason for this has been major difficulties in extracting sufficient DNA from older herbarium specimens that is of good enough quality for use in PCR amplification of the target sequences. Unfortunately the DNA from herbarium specimens is often highly degraded, depending on the conditions of drying and storage [4]. During storage, numerous alterations of the DNA take place, a topic extensively reviewed by Pääbo et al. [5]. The second obstacle with respect to the use of herbarium material has been that many of the specimens collected are dating back to the 19^th^ and early 20^th^ century, thousands of samples were collected for morphological and taxonomic comparison. Many of these specimens are to be considered historic artefacts of great value, and this is especially true for type specimens. Therefore, only minute amounts of material could be taken from the specimens without doing irreparable damage to them. As an example, the Wageningen herbarium generally allows 50 mg (maximum 5%) of a non-type herbarium specimen to be removed (http://www.bis.wur.nl/UK/Links/DNAProtocol/). This equates roughly to a 4 cm^2^ area of a medium thick leaf. During the past ten years, the extraction from herbarium samples has been comparatively investigated several times [6]–[12] and Jankoviak et al. [13] and Walters et al. [14] have reported amplification of up to 500 bp fragments from preserved 100 year old herbarium specimens and seeds respectively. However, in most comparative studies, roughly 100 mg of the specimens were used. With respect to herbarium vouchers of small plants or fungi, and especially phytopathogenic Oomycota and Eumycota, taking this amount of material would often result in the complete destruction of the specimens. For phytopathogenic species it is not uncommon that only minor parts of the specimens are affected by the disease and often herbarium specimen consist only of a single leaf. Therefore it was the aim of this study to test a variety of DNA extraction protocols (19 protocols, including modifications) and DNA polymerases (15 tested) to evaluate the best-suited method to extract and to amplify DNA from 2 mg of up to 130 years old preserved leaf tissue infected with oomycete pathogens.

## Results

**Figure 1:**
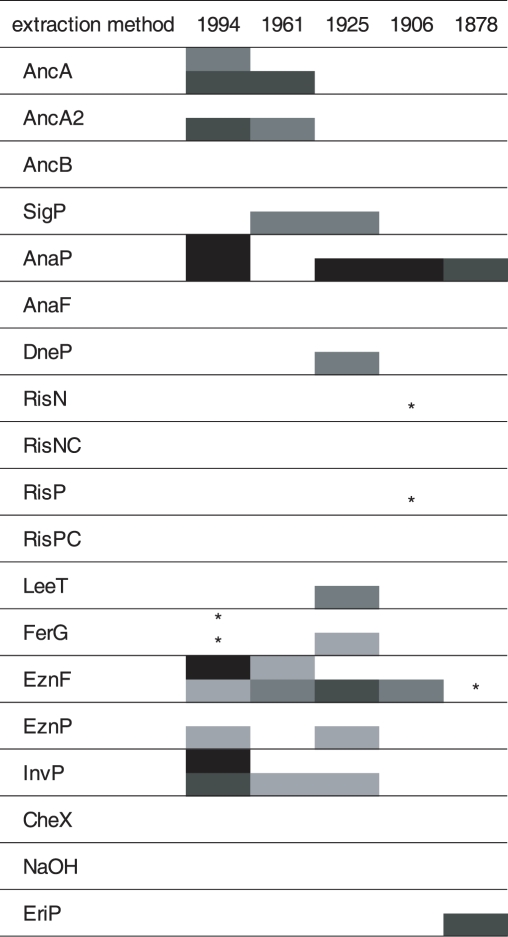
Results of the PCR amplification of cox2 fragments from up to 129 years old herbarium specimens (leaves infected by biotrophic oomycete pathogens), using an amount of DNA equalling 0.05 mg of starting material. Black fields: amplicon amount >90 ng, dark grey: amplicon amount 30–90 ng, grey: amplicon amount 10–30 ng, light grey: amplicon amount <10 ng, white: no amplicon detectable, asterisks: very faint band visible (≪10 ng). Upper half of each row: ∼620 bp fragment, lower half: ∼350 bp fragment.

The 19 different DNA extraction methods yielded highly diverging amounts of DNA, as presented in Table 1. In all cases, the DNA was highly fragmented and gave a smear on agarose gels, revealing mostly fragment sizes below 500 bp (data not shown). DNA extracts were colourless to brownish, depending on the method used. All DNA-amplicons sequenced revealed the target sequence and no contamination was observed. In an initial test, an amount of DNA extracted which equalled 0.05 mg of dried plant material was used in the PCR reactions (Fig. 1). Only 13 DNA extraction protocols gave PCR amplicons for one or more specimens. From these, 3 DNA extracts (gained by the methods AnaP, EznF, EriP) gave a 350 bp amplification product from a 129 year old sample.

**Table 1:** Total DNA amount, OD* values at 260 nm and standard quality scores per sample and extraction method.

extraction method	elution volume	1994				1961				1925				1906				1878			
		µg	OD_260 nm_	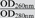	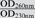	µg	OD_260 nm_	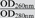	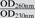	µg	OD_260 nm_	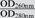	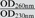	µg	OD_260 nm_	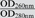	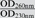	µg	OD_260 nm_	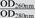	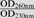
AncA	100 µl	**1.94**	0.388	2.32	0.03	**6.56**	1.313	2.25	0.07	**4.51**	0.902	2.20	0.05	**2.82**	0.565	2.08	0.03	**5.70**	1.139	2.18	0.07
AncA2	100 µl	**2.34**	0.469	3.48	0.03	**5.53**	1.106	2.40	0.08	**2.41**	0.482	2.75	0.03	**4.06**	0.812	1.75	0.05	**3.61**	0.722	1.87	0.04
AncB	100 µl	**2.71**	0.542	1.31	0.03	**2.26**	0.451	2.56	0.04	**3.18**	0.636	1.52	0.04	**3.27**	0.654	1.42	0.04	**3.13**	0.627	1.58	0.06
SigP	100 µl	**0.49**	0.098	3.26	0.35	**0.66**	0.132	3.42	0.50	**0.47**	0.094	1.80	0.35	**0.42**	0.084	2.05	0.26	**0.39**	0.078	6.71	0.10
AnaP	200 µl	**2.84**	0.284	1.72	0.08	**6.96**	0.696	1.93	0.31	**3.53**	0.353	2.07	0.70	**2.56**	0.256	1.90	0.39	**3.28**	0.328	2.03	0.10
AnaF	30 µl	**6.09**	4.063	1.76	0.24	**15.20**	10.131	1.94	0.74	**9.98**	6.652	1.77	0.57	**7.69**	5.125	1.82	0.44	**8.82**	5.882	1.88	0.63
DneP	100 µl	**1.04**	0.208	1.60	0.84	**1.16**	0.232	1.75	0.92	**0.76**	0.152	1.43	0.71	**0.58**	0.116	3.71	0.65	**0.70**	0.141	1.49	0.59
RisN	40 µl	**6.87**	3.436	1.75	1.01	**10.06**	5.032	1.83	1.32	**9.16**	4.582	1.96	0.99	**6.75**	3.375	2.15	0.83	**9.46**	4.729	1.88	1.54
RisNC	100 µl	**8.72**	1.744	1.75	0.94	**8.22**	1.645	1.85	1.02	**8.95**	1.790	1.82	0.97	**7.05**	1.411	1.90	0.85	**12.20**	2.441	1.83	1.40
RisP	40 µl	**4.61**	2.304	1.92	1.39	**11.75**	5.877	1.90	1.39	**6.60**	3.300	2.00	1.24	**6.55**	3.277	2.03	0.84	**11.34**	5.670	1.81	1.44
RisPC	100 µl	**8.11**	1.622	1.84	1.27	**10.21**	2.042	1.85	1.17	**9.59**	1.917	1.94	1.21	**7.63**	1.526	1.84	0.89	**11.34**	2.267	1.86	1.43
LeeT	40 µl	**6.65**	3.324	1.87	1.47	**16.71**	0.835^a^	1.87	1.60	**4.80**	2.401	1.96	1.71	**3.85**	1.804	2.00	1.65	**9.80**	4.902	1.92	2.00
FerG	20 µl	**0.07**	0.072	2.14	1.59	**0.08**	0.078	2.08	2.04	**0.14**	0.139	3.22	2.94	**0.04**	0.037	1.88	3.55	**0.11**	0.114	1.88	1.88
EznF	100 µl	**1.43**	0.287	2.31	0.32	**3.57**	0.715	2.25	0.63	**1.31**	0.262	2.09	0.24	**0.65**	0.130	5.67	0.12	**0.10**	0.021	−2.60	0.03
EznP	100 µl	**1.15**	0.229	1.69	0.58	**1.46**	0.293	1.57	0.64	**0.82**	0.163	1.50	0.43	**0.68**	0.135	1.59	0.40	**0.33**	0.067	2.30	0.31
InvP	100 µl	**4.26**	0.852	2.01	0.70	**8.54**	1.708	2.01	1.03	**4.58**	0.917	2.13	0.73	**2.45**	0.490	2.04	0.40	**3.59**	0.717	1.95	0.67
CheX	200 µl	**64.90** b	0.649^a^	1.10	0.36	**59.72** b	0.597^a^	1.29	0.55	**66.48** b	0.665^a^	1.10	0.48	**114.58** b	1.146^a^	0.96	0.49	**73.58** b	0.736^a^	1.11	0.51
NaOH	200 µl	**93.86** b	0.939^a^	0.91	0.34	**78.78** b	0.788^a^	1.31	0.54	**90.82** b	0.908^a^	1.00	0.44	**49.34** b	0.987^a^	0.79	0.42	**48.23** b	0.965^a^	1.12	0.49
EriP	100 µl	**4.61**	0.921	1.87	1.27	**17.78**	3.556	2.05	1.75	**5.13**	1.026	1.83	1.17	**4.55**	0.910	1.69	0.76	**2.68**	0.536	1.75	0.99

* OD_260 nm_ of 1 equals 50 ng/µl.

a dilution 1∶10.

b highly overestimated DNA content due to impure extracts.

**Figure 2:**
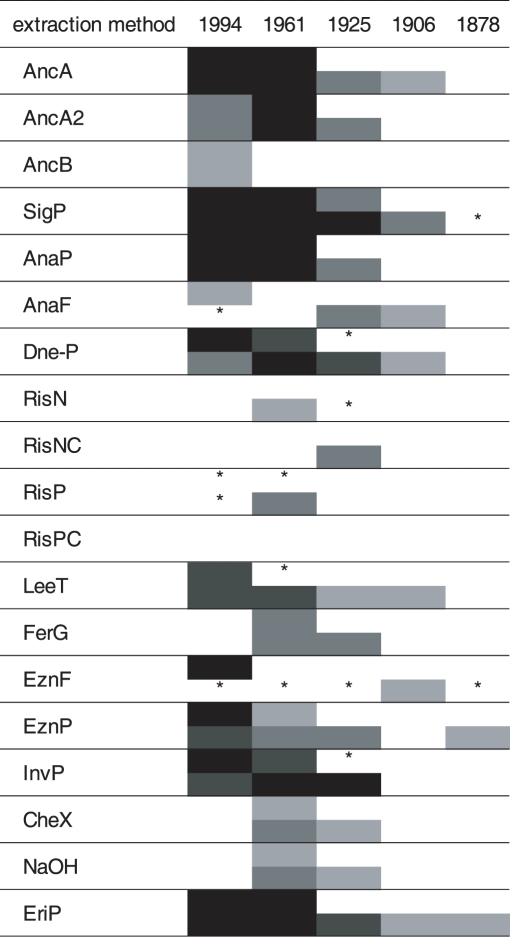
Results of the PCR amplification of cox2 fragments from up to 129 years old herbarium specimens (leaves infected by biotrophic oomycete pathogens), from 10 ng of extracted DNA. Black fields: amplicon amount >90 ng, dark grey: amplicon amount 30–90 ng, grey: amplicon amount 10–30 ng, light grey: amplicon amount <10 ng, white: no amplicon detectable, asterisks: very faint band visible (≪10 ng). Upper half of each row: ∼620 bp fragment, lower half: ∼350 bp fragment.

When using 10 ng of extracted DNA per reaction (Fig. 2), eight protocols gave only PCR amplicons of the *cox2* region for one or two of the specimens, and with none of these protocols (AncB, RisN, RisNC, RisP, Ris PC, FerG, CheX, NaOH), amplification of the target sequence was obtained for samples more than 100 years old. Only the DNA extracts from five protocols gave 350 bp amplification products for the 129 year old sample (SigP, AnaP, EznF, EznP, EriP) in either absolute (i.e. extract amount) or relative (i.e. specific amount in ng) PCR. The larger fragment (∼620 bp) could only be amplified from the samples less than 100 years old, when using the Fermentas Taq DNA polymerase. It was observed that with increasing age of the samples PCR amplification performance decreased.

**Figure 3:**
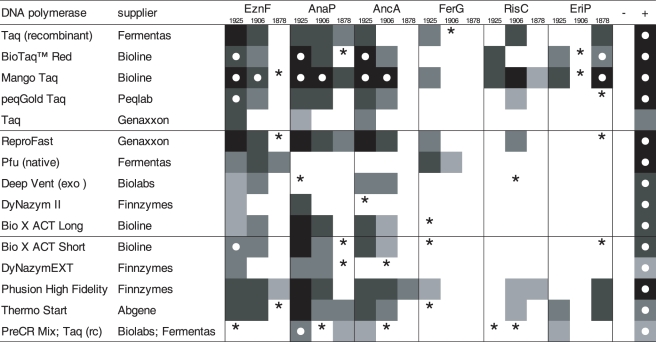
Comparison of the performance of various DNA polymerases. Square fields indicate amplification of the ∼350 bp fragment with different intensity. Black fields: amplicon amount >90 ng, dark grey: amplicon amount 30–90 ng, grey: amplicon amount 10–30 ng, light grey: amplicon amount <10 ng, white: no amplicon detectable, asterisks: very faint band visible (≪10 ng); white dots indicate additional amplification of the ∼620 bp fragment. +: positive control, −: negative (water) control.

Of the DNA extraction methods applied in this study, four gave consistent *cox2* amplification throughout all samples tested (SigP, AnaP, EznF, EriP). For subsequent experiments, three of these were selected, representing different methodologies (AnaP, EznF, EriP). To these, the extraction method of May and Ristaino (15: RisNC), which was reported to be suitable for amplification from minute amounts of a more than 150 year old herbarium specimen, a commercial DNA extraction kit especially designed for extraction of ancient DNA (AncA), and a simple genomic DNA extraction kit (Fermentas, FerG) were added. The DNA obtained by these protocols from the three oldest samples (82, 101, 129 years) was used to test the amplification performance of 15 DNA polymerases, encompassing 5 conventional Taq polymerases, one hot-start Taq polymerase, a DNA- Repair Kit, 3 Taq polymerase based blends, 8 proofreading enzymes, and a genetically engineered DNA polymerase. All polymerases tested gave amplification in the positive control, which was represented by a herbarium specimen less than three years old. For the herbarium specimens tested, the DNA polymerases tested showed highly different amplification performances (Fig. 3). Only four of the polymerases tested yielded the ∼620 bp fragment from at least one of the DNA extracts obtained by at least one DNA protocol (BioTaqRed, Mango-Taq, peqGoldTaq, BIO-X-ACT short). In addition, also the PreCR-mix gave amplification of the ∼620 bp fragment for one of the DNA extracts tested. Only two polymerases were able to amplify the ∼620 bp fragment from the oldest sample (BioTaq Red, Mango-Taq). The best performance was exhibited by the Mango-Taq DNA polymerase, which was the only polymerase which was able to amplify the ∼620 bp amplification product from the 102 year old sample.

A cross comparison of DNA extraction methods and DNA polymerase used revealed that the DNA extracts (10 ng of DNA per PCR reaction) gained from the Analytikjena DNA extraction kit (AnaP) were suitable for the use of most polymerases, except for two enzymes. Also the DNA extracted by means of the E.Z.N.A. forensic kit (EznF) was amplifiable by a wide range of polymerases, including proofreading enzymes. The extraction method using N-phenacylthiazolium bromide (EriP) did not yield DNA consistently amplifiable by a wide range of polymerases, but when using Mango-Taq (Bioline) or BioTaq Red (Bioline), the ∼620 bp fragment could be obtained from the oldest herbarium sample. DNA obtained from the May and Ristaino protocol (15: RisNC) did not yield optimal results, and dilution resulted in a decrease of PCR product (data not shown). In general, AnaP and EriP, in combination with using Mango-Taq were giving best results.

## Discussion

The use of ancient and historic DNA is becoming ever more popular since the first major breakthroughs in amplifying DNA from Egyptian mummies [16], mammoths [17] and Neanderthals [18]. For a time, it seemed like there would be virtually be no age limit for the survival of DNA [19]. However, more often than not it later turned out that reports of antediluvian DNA were the result of contamination with more recent DNA (e.g. 20). However, regarding the success with animal remains [21], archeological olive pits [22] and a well preserved coprolite from the Miocene [23], it seems odd that it still is considered a major challenge to obtain DNA from herbarium samples only a few decades old. The reason for this is probably the suboptimal drying and storage conditions for many historical herbarium specimens, which results in DNA modification and degradation [24], [25]. But as herbarium specimens constitute an invaluable resource for the study of genetic diversity of plants, algae and fungi, several comparative studies have addressed the problem of extracting DNA from herbarium specimens. In most cases, only few different DNA extraction protocols were applied [8], [10], [11], [12], [26], with the notable exception of Li et al. [9]. This is regrettable, considering the importance of the subject. The most successfully applied methods were either CTAB-based methods [6], [27], or commercially available DNA extraction kits [8]. Especially the DNeasy plant extraction kit (DNeasy Plant Mini Kit, Qiagen) in particular has proved to be the best for the extraction of DNA from historic specimens [8], [22]. Also in our study, this kit was amongst the best of the commercially available kits tested, with only two other kits performing slightly better (AnaP, SigP) on the oldest samples.

In most cases, roughly 100 mg of plant material were used. Also Li et al. [9] used relatively high amounts of plant tissue and there was no standardized starting material for each of the protocols they tested. In their latest study [28], 30 mg of plant material were used, which although a great improvement, still constitutes major loss of valuable tissue, especially in smaller specimens. Therefore it was an important aim of this study to test DNA extraction methods on smaller amounts of plant tissue, and we chose to extract DNA from 2 mg dry weight of starting material, which is 15 times less than the amount used by Li et al. [28]. As Ristaino et al. [29] had previously reported the successful amplification from a more than 150 year old specimen from the Irish Potato Famine, from only few square millimetres of infected leaf tissue, this method was tested on the specimens used by us, also with several modifications. Our investigations did not yield similarly promising results with this method, and although the smaller *cox2*-fragment could be obtained when using the best-suited polymerase, other DNA extraction methods performed significantly better. Also further dilution of the extracts, as done by May and Ristaino [15], did not improve the results but resulted in decreasing amplicon amounts until complete loss. Therefore, it might be appropriate to resample the specimens investigated by Ristaino [9] and May and Ristaino [15], using additional phylogenetic markers in order to track historic epidemics of *Phytophthora infestans* in more detail.

Very recently, Lister et al. [30], reported that the combination of PTB and a commercial DNA purification kit yielded results superior to previous methods and was successfully applied to amplify a 350 bp fragment from an a specimen that was over a 100 years old. The amount of material used was 10–40 mg, but amplification could only be obtained from grains and not from leaf blades, most likely due to inhibitory substances in the DNA extracts. Nonetheless, the progress reported by Lister et al. [30] in comparison to previous studies is obvious and it seems likely that this success was mainly due to the application of PTB, which has been successfully used in studies investigating Miocene ground sloth coprolite [23] and processed wood [26]. Adding PTB to the lysis buffer of the Analyticjena plant DNA extraction kit (final concentration of 2.5 mM) for other samples than those processed for this comparative study, provided a quick and reliable kit-based extraction protocol for the recovery of amplifiable ancient DNA. This protocol has resulted in the amplification of the ∼620 bp fragment for samples more than 130 years old (Thines et al., in preparation).

In none of the studies focussing on improving the extraction of DNA from herbarium specimens, were the resulting DNA extracts tested with a variety of DNA polymerases. This study clearly demonstrates that not only the DNA extraction protocol used but also the choice of polymerase greatly influences the chances of getting successful PCR amplification. Apart from different enzyme performance, this is most likely due to the purity of the enzymes and the chemistry of the polymerase buffers supplied along with the enzymes. Considering that it was only possible to test a fraction of the plethora of DNA polymerases that are currently promoted by numerous companies, it is obvious that the full potential of DNA polymerase comparison for amplification from historic herbarium specimens is far from being fully exploited.

With respect to the historical, cultural and scientific value of herbarium specimens, additional studies are required to further minimize the amount of material necessary for reliable PCR-amplification. This would potentially enable scientists to exploit the ‘untapped genetic treasure trove’ that is currently in thousands of herbaria worldwide. This is especially crucial for plant parasites, which are easily distributed because of the global trade in seeds and plants and pose a major thread for horticulture and agriculture.

## Materials and Methods

### Preparation of the plant material and DNA extraction

In total, 50–100 mg of infected plant tissue from herbarium voucher specimens (Table 2) were taken and disrupted in a mixer mill (MM2, Retsch, Germany), using six iron balls of 3 mm diameter per sample. The powdered tissue was than portioned to 2 mg portions in 2 ml reaction tubes (Sarstedt, Germany) using a precision gauge (R200D, Sartorius, Germany). 19 reaction tubes could be prepared for each of the samples and were subjected to DNA extraction. Sample preparation and DNA extractions were carried out in a contaminant free environment. No recent samples of the organisms investigated were processed in the laboratory before or during the experiments reported here.

**Table 2:** Herbarium vouchers investigated.

pathogen	host	year	herbarium^1^ accession number	collector	location/country
*Sclerospora graminicola*	*Setaria verticillata*	1878	BR 82377246	Thümen, F.	Parma, Italy
*Sclerospora graminicola*	*Setaria viridis*	1906	BR 8237623	Magnus, P.W.	Brixen, Germany
*Sclerospora graminicola*	*Setaria viridis*	1925	BR 82373200	Sydow, H.	Tamsel, Germany
*Albugo sp.*	*Reseda sp.*	1961	BP 3942		
*Sclerospora graminicola*	*Pennisetum glaucum*	1994	HOH HUH sg048	Thakur, R.P.	Myosore, India

1 BR: National Botanic Garden of Belgium, BP: Hungarian Natural History Museum, HOH: Herbarium of the University of Hohenheim.

The DNA extraction protocols used are summarized in Table 3, with the manufacturers given there. All DNA extractions were carried out exactly as given in the respective references or according to the manufacturer's instructions in case of commercially available DNA extraction kits. The resulting DNA was eluted in sterile water (DEPC-treated, Carl Roth, Germany).

**Table 3:** List of protocols used for DNA extraction.

internal code	method or kit name	reference or supplier (catalogue no.)
AncA	GENECLEAN® Kit for Ancient DNA	BIO 101
AncA2	GENECLEAN® Kit for Ancient DNA	BIO 101
AncB	GENECLEAN® Kit for Ancient DNA	BIO 101
SigP	GenElute™ Plant Genomic DNA Miniprep Kit	Sigma (G2N10)
AnaP	innuPREP Plant DNA Kit	Analytikjena (845-KS-10600)
AnaF	innuPREP Forensic Kit (Protocol 7)	Analytikjena (845-KS-10500)
DneP	Dneasy Plant Mini Kit	Qiagene (69104)
RisN	CTAB DNA extraction	Ristaino et al. 2001 [29]
RisNC	CTAB DNA extraction with column	May and Ristaino 2004 [15]
RisP	CTAB/PVP DNA extraction	modification of Ristaino et al. 2001 [29]
RisPC	CTAB/PVP DNA extraction with column	modification of May and Ristaino 2004 [15]
LeeT	DNA extraction Lee & Taylor	Lee and Taylor 1990 [31]
FerG	Genomic DNA Purification Kit	Fermentas (#K0519)
EznF	E.Z.N.A.™ Forensic DNA Isolation Kit	VWR (D3591-00)
EznP	E.Z.N.A.™ Plant DNA Isolation Kit	VWR (D3485-00)
InvP	Invisorb® Spin Plant Mini Kit	Invitek (10371002)
CheX	Chelex® 100 DNA extraction	Walsh et al. 1991 [32]
NaOH	alkaline lysis	Klintschar and Neuhuber 2000 [33]
EriP	Ancient DNA extraction	Erickson et al. 2005 [34]

The kit for ancient DNA extraction was carried out three times exactly as given in the manual with the three different, supplied lysis solutions (AncA, AncA2, AncB, respectively), and DNA was eluted in 100 µl water. In the SigP extraction method the longer drying variant was chosen. The lysis incubation step was carried out for 25 min in the EznP method. In the genomic DNA kit (Fermentas, Germany) the note for small amounts of DNA was followed, including precipitation for 20 hours and dissolving the DNA obtained in 20 µl water. The RisN method [29] was preformed with doubled extraction volume. In a modified procedure, 5% polyvinylpyrolidone (PVP) was added to the nuclei lysis buffer (RisP). Both procedures were also modified according to May and Ristaino (15: RisNC, RisPC) with the column based purification and concentration step reported there.

DNA concentration was measured using a spectrophotometer for small volumes (NanoDrop ND-1000, NanoDrop Technologies, USA).

### PCR and sequencing

For PCR-amplification of the mitochondrial cytochrome oxidase subunit II (*cox2*) gene region modified primers and PCR conditions of Hudspeth et al. [35] were used. For all experiments, a single tube semi-nested approach was chosen, using forward (3′-GGCAAATGGGTTTTCAAGATC-5′), reverse (3′-CCATGATTAATACCACAAATTT-5′) and the forward nested (3′-GGTAGTCAATGGTATTGG-5′) primer in a single reaction. All of these primers are oomycete specific and would not amplify human, plant, fungal or bacterial DNA. Negative and positive PCR controls were done for all experiments reported in this study. To ensure maximum comparability, the PCR data presented is based on the same chemical background where possible, i.e. a single PCR mastermix was used were applicable. In addition, PCR reactions were carried out in only two PCR runs on the same thermal cycler (Primus 96, Peqlab, Germany).

For the initial comparisons, the standard recombinant Taq DNA polymerase from Fermentas (Fermentas, Germany) was used with magnesium chloride and ammonium sulphate 10× PCR buffer supplied by the manufacturer. Apart from PCR-buffer, PCR reactions contained 0.2 mM dNTPs (Fermentas, Germany), 2.0 mM MgCl_2_, 1.0 µM of each primer (Sigma-Aldrich, Germany) and 1.2 units of Taq DNA polymerase per 30 µl. PCR cycling conditions were as follows: 4 min at 94°C initial denaturation; 36 cycles of 40 s at 94°C, 40 s at 51°C, 1 min at 72°C; and a final extension of 4 min at 72°C.

For assessing the best suited DNA polymerase to amplify the *cox2* region of the pathogens from the oldest herbarium specimens, the 15 polymerases listed in Fig. 3 were applied according to the manufacturer's instructions, using 1 unit of polymerase and the specific polymerase buffers supplied in 30 µl reactions with the same cycling conditions as mentioned before except for an additional 15 min, 94°C activation step in the hot-start DNA polymerase tested.

Amplicon confirmation by sequencing was carried out by a commercial sequencing company (GATC, Germany) using the modified cox2-R primer.
